# Functional Thresholds Derived from Dynamometry and 6-Minute Walk Test with Morphofunctional Assessment to Guide Individualized Exercise Prescription in Cardiac Rehabilitation

**DOI:** 10.3390/jcm15114336

**Published:** 2026-06-03

**Authors:** María del Mar Amaya-Campos, Ramón Zafra Jiménez, Rocío Fernández-Jiménez, Isabel M. Vegas-Aguilar, María García-Olivares, Mónica Diaz-Cordovés Rego, Yolanda Ruiz Molina, Adela María Gómez González, Angel Montiel Trujillo, Francisco Tinahones-Madueño, José Manuel García-Almeida, Lucía Jiménez Laguna

**Affiliations:** 1 Department of Endocrinology and Nutrition, Virgen de la Victoria University Hospital, 29010 Malaga, Spain; mariadelmarac@ibima.eu (M.d.M.A.-C.); rocio.fernandez.j@juntadeandalucia.es (R.F.-J.); isabe.mva.13@ibima.eu (I.M.V.-A.); maria.olivares@ibima.eu (M.G.-O.); fjtinahonesmadueno.sspa@juntadeandalucia.es (F.T.-M.); josem.garcia.almeida.sspa@juntadeandalucia.es (J.M.G.-A.); 2 Instituto de Investigación Biomédica y Plataforma en Nanomedicina, Málaga Biomedical Research Institute and BIONAND Platform, 29010 Malaga, Spain; 3 Department of Medicine and Dermatology, Málaga University, 29016 Malaga, Spain; luciajimenezlaguna@gmail.com; 4 Endocrinology and Nutrition Unit, Son Espases University Hospital, 07120 Palma de Mallorca, Spain; 5 Department of Endocrinology and Nutrition, Quiron Salud Málaga Hospital, Av. Imperio Argentina, 29004 Malaga, Spain; 6 Rehabilitation Medicine Unit, Virgen de la Victoria University Hospital, 29010 Malaga, Spain; mariam.diazcordoves.sspa@juntadeandalucia.es (M.D.-C.R.); yolanda.ruiz.sspa@juntadeandalucia.es (Y.R.M.); 7 Department of Cardiology, Virgen de la Victoria University Hospital, 29010 Malaga, Spain; angel.montiel.sspa@juntadeandalucia.es; 8 Centro de Investigación Biomédica en Red de Fisiopatología de la Obesidad y Nutrición (CIBEROBN), Carlos III Health Institute (ISCIII), University of Málaga, 29016 Malaga, Spain

**Keywords:** cardiac rehabilitation, exercise prescription, handgrip strength, six-minute walk test, bioelectrical impedance analysis, nutritional ultrasound, body composition, muscle strength, functional assessment

## Abstract

**Background/Objectives**: To evaluate the associations and concurrent validity between baseline functional and morphofunctional assessments in patients with cardiovascular disease participating in a Phase II cardiac rehabilitation program, as a basis for informing individualized exercise prescription. **Methods**: We conducted an observational retrospective cross-sectional study of patients enrolled in a Phase II outpatient cardiac rehabilitation program (January 2021–December 2023, Málaga). Functional assessments included handgrip strength (HGS), isometric biceps and quadriceps dynamometry, and direct assessment of 20-repetition maximum (20RM) through dynamic resistance exercises using external loads (defined as the maximum load allowing approximately 20 repetitions to near muscular fatigue). Aerobic capacity was evaluated using the 6-min walk test (6 MWT) and a modified Bruce exercise stress test with estimated METs. Morphofunctional assessment included vector bioimpedance analysis (phase angle [PhA], fat-free mass [FFM], body cell mass [BCM]) and rectus femoris ultrasound (cross-sectional area [RF-CSA] and contracted diameter [RF-CON]). Correlation and linear regression analyses were performed. **Results**: The sample included 223 participants (78.0% male; age 57.7 ± 8.6 years). HGSmax correlated strongly with 20RM biceps (r = 0.89) and moderately with quadriceps (r = 0.72). 6 MWT distance and speed correlated with ergometry-derived METs (r = 0.38–0.40; *p* < 0.001), whereas Borg ratings correlated inversely with METs and exercise time (r = −0.32 to −0.34; *p* < 0.001). PhA, BCM, FFM, and rectus femoris ultrasound measures correlated with both strength and aerobic outcomes (ρ ≈ 0.33–0.50; all *p* < 0.001). In regression analyses, HGSmax was the main predictor of 20RM biceps (R^2^ = 0.792) and showed moderate predictive capacity for quadriceps performance (R^2^ = 0.521). The MET model demonstrated limited explanatory capacity (R^2^ = 0.288). **Conclusions**: The integration of simple, accessible, and reproducible tools such as HGS and the 6 MWT with morphofunctional parameters may provide a pragmatic approach to support individualized exercise prescription in cardiac rehabilitation. While stronger associations were observed for upper-limb resistance performance, the predictive capacity for lower-limb strength and aerobic exercise intensity was more moderate and should be interpreted cautiously. These findings support the potential clinical utility of combining functional and morphofunctional assessments in routine cardiac rehabilitation practice.

## 1. Introduction

Cardiovascular diseases (CVD) remain the leading cause of death worldwide [1]. Major cardiovascular risk factors include obesity, hypertension, type 2 diabetes, smoking, and physical inactivity [2,3,4,5,6,7]. Beyond acute management, long-term care requires effective secondary prevention strategies aimed at improving functional status, reducing recurrent events, and promoting sustained lifestyle change.

Cardiac rehabilitation (CR) is a comprehensive intervention that integrates cardiovascular risk factor management, structured exercise training, health education, and psychosocial support [8]. It is commonly delivered in three phases: an inpatient phase focused on clinical stabilization and early mobilization, an outpatient phase centered on supervised exercise and secondary prevention, and a maintenance phase aimed at preserving long-term adherence to healthy behaviors [9]. CR has consistently been associated with improvements in functional capacity, quality of life, cardiovascular risk factor control, and clinical outcomes, while also representing a safe and cost-effective intervention [8,9,10,11].

Cardiac rehabilitation and individualized exercise prescription are related but distinct concepts; while cardiac rehabilitation represents a structured, multidisciplinary intervention, individualized exercise prescription involves the tailored adjustment of training variables according to the patient’s clinical condition, functional capacity, and disease severity, particularly in heterogeneous populations such as those with heart failure.

Exercise prescription is one of the core components of CR, and current guidelines recommend combining aerobic and resistance training to maximize clinical benefit [12,13,14,15,16]. However, individualized prescription requires an adequate assessment of both muscular strength and cardiorespiratory fitness. For resistance training, one-repetition maximum testing is one of the best validated methods for strength assessment [17], but its routine use may be limited in patients with CVD because of safety concerns, equipment requirements, or time constraints. Submaximal approaches, such as estimated 20-repetition maximum (20RM), may offer a more feasible alternative in cardiac populations. Accordingly, the use of 20RM was preferred over 1RM in this population, as it allows the estimation of muscular performance using submaximal loads, thereby reducing excessive hemodynamic stress while prioritizing sustained muscular effort relevant to functional capacity in cardiac rehabilitation [12,18]. In this context, handgrip strength has emerged as a simple, safe, and reproducible measure that is associated with nutritional status, functional performance, and clinical outcomes [19]. For aerobic prescription, exercise stress testing remains the reference method for estimating metabolic equivalents (METs) and exercise tolerance [20], although simpler field-based tools are often needed in routine practice. The 6-min walk test (6 MWT) is a feasible and reliable option in patients with heart disease and provides clinically relevant information on functional capacity and prognosis [21].

In parallel, nutritional and body composition assessment in CR has evolved beyond conventional anthropometric and biochemical measures. Morphofunctional assessment integrates structural and functional parameters, including bioelectrical impedance analysis, muscle strength, and nutritional ultrasound, to provide a more comprehensive evaluation of body composition, muscle mass, and muscle quality [22,23,24]. These approaches may facilitate early detection of muscle impairment, improve risk stratification, and support more personalized therapeutic interventions. Nevertheless, their practical value for guiding individualized exercise prescription in outpatient CR remains insufficiently defined.

Therefore, the aim of this study was to explore the associations between baseline functional and morphofunctional assessments and functional parameters related to individualized resistance and aerobic exercise prescription in patients with cardiovascular disease participating in a Phase II cardiac rehabilitation program.

## 2. Materials and Methods

### 2.1. Study Design and Setting

We conducted an observational, retrospective, cross-sectional study of patients enrolled in the phase II outpatient cardiac rehabilitation program at Virgen de la Victoria University Clinical Hospital (Málaga, Spain) between January 2021 and December 2023.

The program consisted of three supervised 1 h sessions per week, including a 10 min warm-up, 20 min of resistance training targeting major muscle groups, 20 min of aerobic exercise (treadmill or cycle ergometer), and 10 min of stretching and relaxation. Exercise intensity was individually prescribed using the Karvonen method, with a target range corresponding to 50–70% of maximal heart rate. Maximal heart rate was defined as the peak heart rate achieved during the exercise stress test rather than age-predicted equations, due to the limited accuracy of theoretical formulas in cardiac populations.

Sessions were supervised by physiotherapists and rehabilitation physicians, with monitoring of heart rate, pulse, and oxygen saturation. In addition, participants attended group-based nutritional education sessions led by dietitians in accordance with the recommendations of the Spanish Society of Cardiology, as well as monthly group psychological support sessions.

Participants were enrolled in a Phase II cardiac rehabilitation program following a cardiovascular event or intervention. The study population predominantly included patients with ischemic heart disease (post-myocardial infarction or angina) who had undergone percutaneous coronary intervention or coronary artery bypass grafting, as well as patients after valve repair or replacement. Patients with both preserved and reduced left ventricular ejection fraction were included, reflecting a heterogeneous cardiovascular population representative of routine clinical practice.

Eligible participants were adults aged 18–80 years who were clinically stable and able to tolerate exercise training. Exclusion criteria were unstable heart failure, acute or inflammatory cardiovascular conditions, severe structural heart disease, uncontrolled arrhythmias, decompensated medical conditions, inability to perform physical exercise, severe psychiatric disorders, age outside the predefined range, or life expectancy of less than 1 year. All participants provided written informed consent.

### 2.2. Clinical, Anthropometric, and Laboratory Variables

Sociodemographic characteristics, anthropometric measurements, cardiovascular risk factors, and relevant clinical history, including ischemic heart disease, prior coronary revascularization, and heart failure, were collected from the medical records. Laboratory data obtained within 3 months before entry into the rehabilitation program were also recorded and included lipid profile parameters and glycemic markers, specifically fasting plasma glucose and glycated hemoglobin (HbA1c).

### 2.3. Lifestyle, Dietary, Quality-of-Life, and Psychological Assessment

Diet quality was assessed using the Prevención con Dieta Mediterránea (PREDIMED) questionnaire. Dietary intake was evaluated using a 72 h food record, including two weekdays and one weekend day, and was analyzed with Dietsource software (version 3.0; Nestlé, Vevey, Switzerland) to estimate daily energy and macronutrient intake.

Physical activity, psychological status, and health-related quality of life were assessed using the International Physical Activity Questionnaire–Short Form (IPAQ-SF), the Hospital Anxiety and Depression Scale (HADS), and the 8-item Short-Form Health Survey (SF-8), respectively.

### 2.4. Morphofunctional Assessment

Morphofunctional assessment included bioelectrical impedance vector analysis (BIVA) and nutritional ultrasound of the rectus femoris muscle.

BIVA was performed using a BIA 101 impedance analyzer (Akern Srl, Florence, Italy) under standardized conditions, including fasting and avoidance of physical exercise in the preceding hours. The recorded parameters included fat-free mass, fat mass, fat-free mass index, fat mass index, skeletal muscle mass index, resistance, reactance, and phase angle. Phase angle was calculated as arctan (Xc/R) × (180/π). A standardized phase angle (SPhA) was derived using age- and sex-specific reference values described by Barbosa-Silva et al. [22], allowing normalization of individual phase angle values relative to a reference population. Standardized phase angle has been proposed as a useful tool for comparing patients with different demographic and anthropometric characteristics and may improve interpretation of nutritional and functional status [23]. Technical accuracy was verified using a precision circuit, and in vivo reproducibility showed coefficients of variation of 1–2% for resistance and reactance.

Nutritional ultrasound of the rectus femoris was performed with a 10–12 MHz linear transducer while participants were in the supine position. Measurements were obtained at the lower third of the distance between the anterior superior iliac spine and the superior pole of the patella, avoiding tissue compression. The assessed muscle parameters included cross-sectional area, circumference, transverse diameter, anteroposterior diameter, muscle thickness, contracted diameter, and leg subcutaneous adipose tissue. Abdominal adipose tissue measurements included total abdominal fat, superficial subcutaneous fat, and visceral fat, all measured at the midpoint between the xiphoid process and the umbilicus. Three measurements were obtained for each parameter, and the mean value was used for analysis. All ultrasound assessments were performed by the same trained examiner [24,25].

### 2.5. Functional Assessment

Muscle strength was assessed by dominant-hand grip strength and isometric bi-ceps and quadriceps dynamometry. Upper- and lower-limb 20-repetition maximum (20RM) loads were assessed using submaximal dynamic resistance exercises. The 20RM technique consisted of selecting a load that allowed approximately 20 maximum repetitions, performed continuously or with short rest-pause breaks until completion and close to muscular failure.

Aerobic capacity was evaluated using the 6 MWT, recording walking distance, heart rate at the end of the test, and Borg 0–20 rating of perceived exertion. This evaluation was complemented by a modified Bruce exercise stress test, from which peak metabolic equivalents (METs) were estimated using standard treadmill-based prediction equations derived from exercise duration and workload rather than direct gas exchange measurements. Exercise duration and hemodynamic response were also recorded.

**Figure 1:**
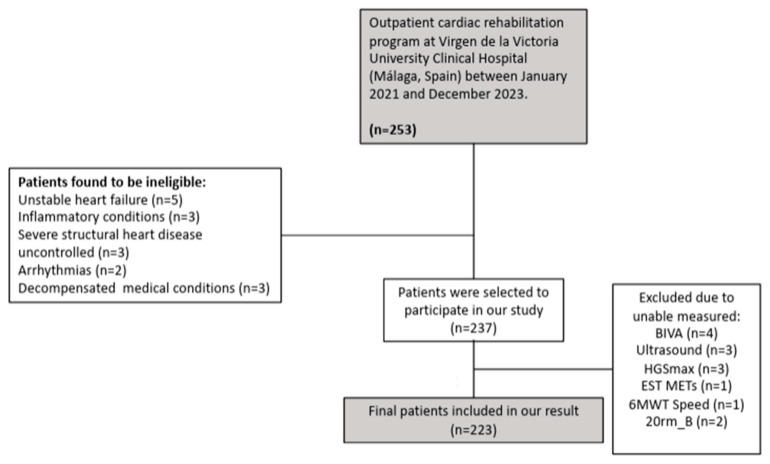
Flowchart of Patient Selection and Assessment Procedures. Patients enrolled in the outpatient cardiac rehabilitation program at Virgen de la Victoria University Clinical Hospital (Málaga, Spain) between January 2021 and December 2023 were screened for eligibility. Exclusion criteria, missing assessments, and the final study population included in the analyses are shown.

Figure 1 summarizes the patient selection process, exclusion criteria, and overall study workflow, including the functional and morphofunctional assessments performed in the cardiac rehabilitation program.

### 2.6. Ethical Considerations

The study was approved by the Málaga Research Ethics Committee (reference number: 2023316125048; code: RECARDIET 2023). Data confidentiality was guaranteed in accordance with Organic Law 3/2018 on Personal Data Protection and Guarantee of Digital Rights and the General Data Protection Regulation (EU) 2016/679.

### 2.7. Statistical Analysis

Statistical analyses were performed using JAMOVI software (version 2.3. Continuous variables were expressed as mean ± standard deviation (SD) for normally distributed data and as median (interquartile range, IQR) for non-normally distributed data. Categorical variables were presented as absolute frequencies and percentages. The normality of continuous variables was assessed using the Shapiro–Wilk test. Associations between functional and morphofunctional variables were examined using Pearson’s or Spearman’s correlation coefficients, as appropriate.

Linear regression models were constructed to estimate 20-RM loads and aerobic exercise intensity, expressed in METs, from clinically accessible and functional variables. All tests were two-sided, and a *p* value < 0.05 was considered statistically significant. Regression model assumptions were evaluated, including normality of residuals, homoscedasticity, and absence of multicollinearity, and were considered acceptable for all models.

### 2.8. Use of Artificial Intelligence

Generative artificial intelligence tools were used exclusively for minor language editing, including grammar, spelling, punctuation, and formatting. No artificial intelligence tools were used to generate scientific content, produce data or graphics, perform statistical analyses, interpret results, or make scientific decisions.

## 3. Results

### 3.1. General Characteristics of the Study Population

A total of 223 participants were included in the study; 78.0% were men, and the mean age was 57.7 ± 8.6 years. Hypertension (84.0%) and dyslipidemia (76.1%) were the most prevalent cardiovascular risk factors, followed by diabetes (27.6%) and obesity (31.9%). According to the International Physical Activity Questionnaire, nearly half of the participants reported low physical activity levels (48.3%), whereas only 4.7% reported high physical activity. Adherence to the Mediterranean diet was moderate in 53.5% of participants, while 16.6% showed high adherence.

Most participants entered the program after catheterization for myocardial infarction (63.8%), followed by angina (15.3%), valve repair or replacement (12.9%), and coronary artery bypass graft surgery (8.0%). Left ventricular ejection fraction was preserved in 73.6% of participants and reduced in 11.7%, and 37.7% were classified as older adults.

Sex-stratified comparisons are shown in Table 1. Significant differences were observed in body size, morphofunctional parameters, including phase angle, fat-free mass index, and rectus femoris cross-sectional area, as well as in strength-related variables, including handgrip strength, biceps and quadriceps dynamometry, and estimated 20-repetition maximum (20-RM) values (*p* < 0.05 for all). No significant differences were found between sexes in age, body mass index, fat mass index, 6 MWT performance, physical activity category, Mediterranean diet adherence, or exercise stress test parameters.

Supplementary Table S1 shows that men had higher body cell mass, skeletal muscle mass index, and rectus femoris dimensions, whereas women had greater leg subcutaneous adipose tissue. Hydration status and sodium-to-potassium ratio did not differ significantly by sex. As shown in Supplementary Table S2, women had higher high-density lipoprotein cholesterol concentrations and lower energy and protein intake, whereas men had higher end-exercise diastolic blood pressure. No other relevant sex-related differences were observed in biochemical, hemodynamic, or quality-of-life variables.

### 3.2. Associations Among Strength, Aerobic Capacity, and Body Composition

**Figure 2:**
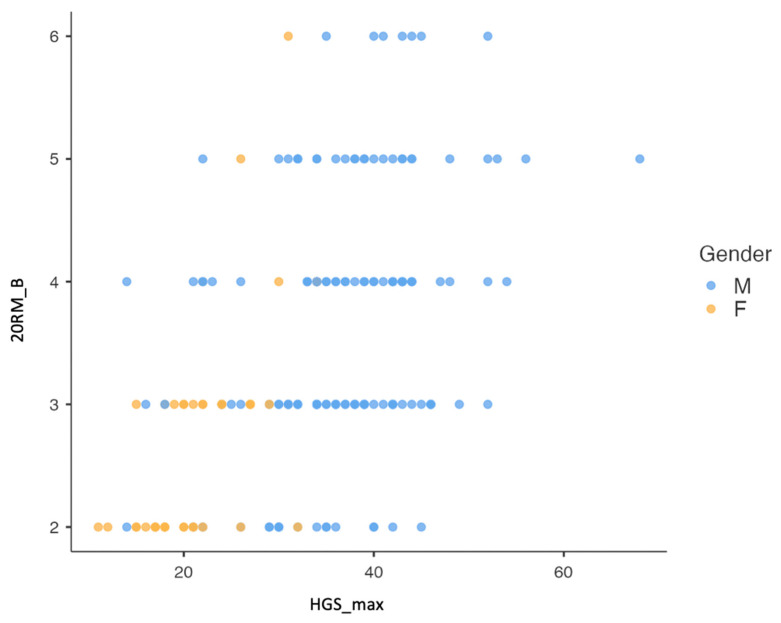
Relationship between maximum handgrip strength and performance in the 20RM biceps test, by sex. Each point represents an individual participant, stratified by sex. The distribution illustrates the association between upper-limb isometric strength and dynamic submaximal performance. Abbreviations: HGSmax, maximal handgrip strength; 20RM_B, 20-repetition maximum biceps; M, male; F, female.

Strong and significant associations were observed among strength-related measures. Maximal handgrip strength correlated strongly with biceps 20-RM (r = 0.89; Figure 2) and moderately with quadriceps 20-RM (r = 0.72). Handgrip strength was also associated with isometric biceps and quadriceps dynamometry (r = 0.56 and r = 0.48, respectively).

Figure 2 illustrates the relationship between maximal handgrip strength (HGSmax) and biceps 20-repetition maximum (20RMB). Individual data points correspond to each participant and are stratified by sex. The figure was constructed to visually explore the association between upper-limb isometric strength, assessed by handgrip dynamometry, and dynamic submaximal strength assessed through direct 20RM testing.

**Figure 3:**
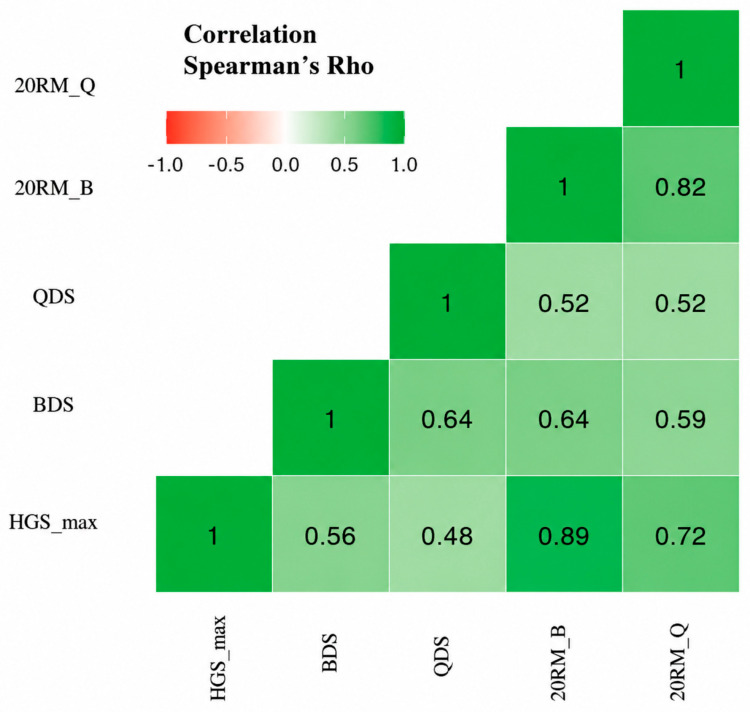
Spearman’s Rho correlation coefficients between different HGSmax and 20RM for upper and lower limbs are shown. Abbreviations: HGSmax = maximum handgrip strength; BDS = Biceps dynamometry strength; QDS = Quadriceps dynamometry strength; 20RM_B = 20 Repetition Maximum biceps; 20RM_Q = 20 Repetition Maximum quadriceps muscle.

In addition, isometric biceps and quadriceps strength showed moderate correlations with both 20-RM measures (r = 0.52–0.64), while biceps 20-RM correlated strongly with quadriceps 20-RM (r = 0.82). Overall internal consistency among strength measures was moderate (Cronbach’s α = 0.729; Figure 3).

**Figure 4:**
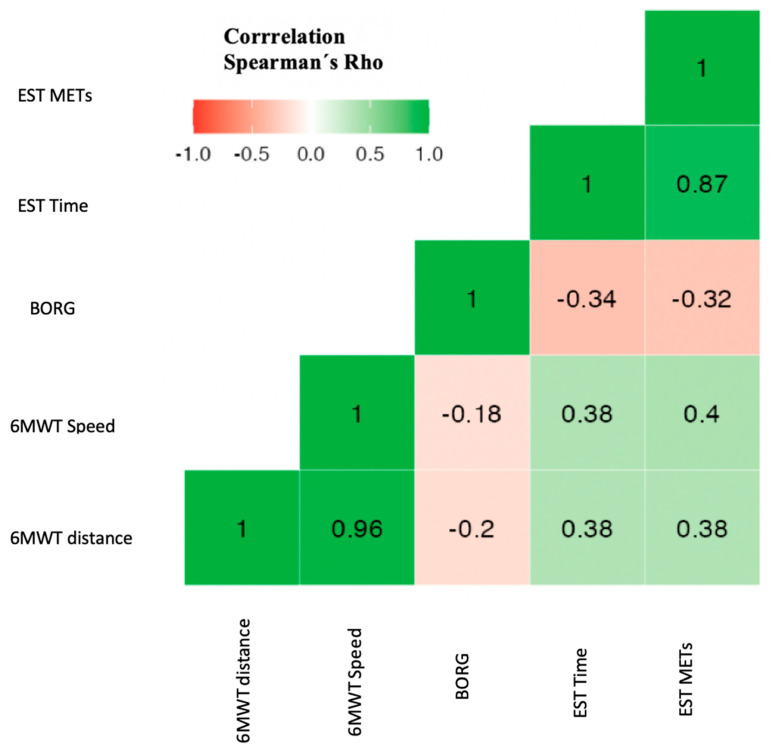
Spearman correlation map rho between the 6 MWT, the EST in terms of duration and METs, and the Borg Rating of Perceived Exertion scale. All displayed correlations were statistically significant (*p* < 0.05). Abbreviations: 6 MWT, 6-min walk test; BORG = Borg scale of perceived exertion; EST = Exercise Stress Test (ergometry); METs: Metabolic Equivalent of Task.

Field- and laboratory-based aerobic measures showed concordant results. Distance covered and walking speed during the 6 MWT correlated positively with ergometry-derived metabolic equivalents (METs) (r = 0.38–0.40; *p* < 0.001) and exercise duration (r = 0.38; *p* < 0.001). By contrast, Borg perceived exertion scores correlated inversely with METs and exercise duration (r = −0.32 and −0.34, respectively; *p* < 0.001 for both). Internal consistency among aerobic measures was moderate (Cronbach’s α = 0.562; Figure 4). Associations between strength and aerobic indices were weaker, including those between handgrip strength and METs (r = 0.22) and between handgrip strength and 6 MWT speed (r = 0.20).

**Figure 5:**
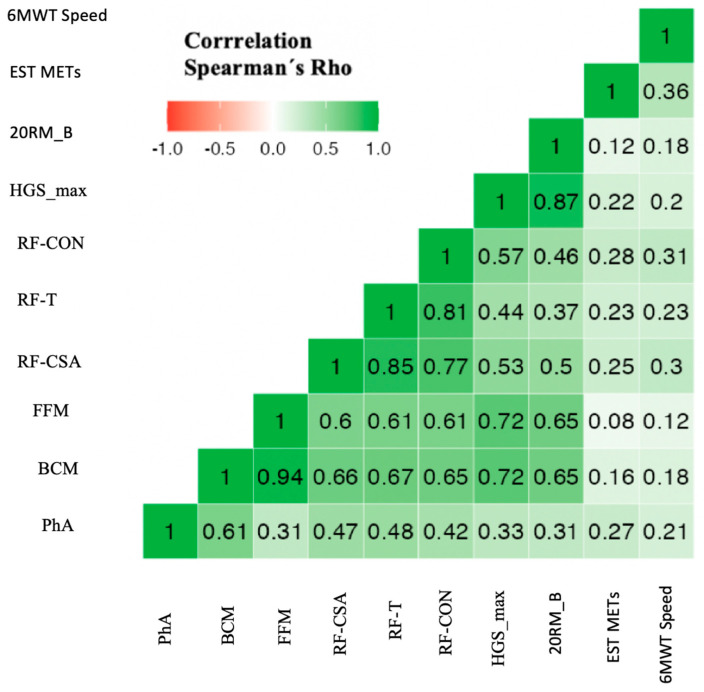
Spearman correlation map rho between morphofunctional parameters obtained through BIVA—including PhA, body cell mass (BCM), and FFM—and nutritional ultrasound variables of the rectus femoris, with functional performance tests such as HGSmax, 20RM_B, EST METs, and 6 MWT speed. All correlations shown were statistically significant (*p* < 0.05). Abbreviations: PhA = Phase Angle; BCM = Body Cell Mass; FFM = Fat-Free Mass; RF-CSA = Rectus Femoris Cross-Sectional Area; RF-T = Rectus Femoris Thickness (*Y*-axis Diameter); RF-CON = Rectus Femoris Thickness during Contraction; HGSmax = Maximum Handgrip Strength; 20RM_B = 20-Repetition Maximum (Biceps); EST METs = Exercise Stress Test in Metabolic Equivalents; 6 MWT Speed = Six-Minute Walk Test Speed.

Parameters derived from vector bioimpedance analysis, including phase angle, body cell mass, and fat-free mass, were consistently and significantly associated with both strength and aerobic performance (ρ ≈ 0.35–0.48; all *p* < 0.001). Likewise, rectus femoris ultrasound variables, particularly cross-sectional area, thickness, and contracted diameter, were correlated with handgrip strength, estimated 20-RM, METs, and 6 MWT (ρ ≈ 0.33–0.50; all *p* < 0.001). The combined functional and morphofunctional measures showed acceptable internal consistency (Cronbach’s α = 0.751; Figure 5).

### 3.3. Regression Models to Estimate Resistance Load and Aerobic Intensity

Linear regression models were developed to estimate upper- and lower-limb 20-RM values from maximal handgrip strength, age, and sex (Table 2). In both models, handgrip strength was the only independent predictor (*p* < 0.001), explaining 79.2% of the variance in estimated biceps 20-RM (β = 0.094; R^2^ = 0.792) and 52.1% of the variance in estimated quadriceps 20-RM (β = 0.131; R^2^ = 0.521). Age and sex were not significant predictors in either model.

Model equations:

Biceps 20-RM = 0.345 + 0.094 × HGSmax − 0.081 × sex + 0.00035 × age

Quadriceps 20-RM = 2.584 + 0.131 × HGSmax + 0.005 × age + 0.003 × sex

A separate regression model was constructed to estimate aerobic exercise intensity, expressed as METs, from age, sex, 6 MWT distance, and Borg perceived exertion score (Table 3). This model showed moderate explanatory capacity (R = 0.536; R^2^ = 0.288). METs increased with greater 6 MWT distance (β = 0.0078; *p* = 0.002) and decreased with older age (β = −0.0817; *p* = 0.001) and higher Borg scores (β = −0.5348; *p* = 0.005). Sex was not a significant predictor (*p* = 0.131).

The derived equation for estimating aerobic exercise intensity was as follows:

METs = 18.49 − 0.0817 × age − 0.7446 × sex (female = 1) + 0.0078 × 6 MWT distance − 0.5348 × Borg rating

## 4. Discussion

The present study supports the integration of functional and morphofunctional assessments as a complementary framework to inform individualized exercise prescription in cardiac rehabilitation. Our findings suggest that simple, accessible tools such as handgrip dynamometry and the 6-min walk test (6 MWT), when interpreted alongside bioimpedance-derived parameters and nutritional ultrasound, may reflect relevant aspects of muscular and aerobic performance in clinical practice.

From a physiological perspective, the observed association between handgrip strength and 20RM performance may be explained by shared determinants of global muscular function. Handgrip strength, although a localized isometric measure, has been widely proposed as a surrogate marker of overall muscle strength, reflecting neuromuscular activation, motor unit recruitment, and general physical conditioning. This is consistent with previous literature describing handgrip strength as a “vital sign” of health (Vaishya et al. [19]) and with studies linking maximal or near-maximal strength to submaximal performance (Grgic et al. [17]; Nuzzo et al. [18]).

However, the differences observed between upper- and lower-limb performance warrant further consideration. The stronger association between handgrip strength and upper-limb 20RM compared with lower-limb performance is physiologically plausible, as upper-limb measures may share more direct neuromuscular and biomechanical determinants. In contrast, lower-limb strength is influenced by additional factors, including locomotor function, postural control, habitual physical activity, and muscle mass distribution. This may explain the moderate predictive capacity observed for quadriceps 20RM and highlights that these measures should not be considered interchangeable, but rather complementary. This interpretation aligns with previous literature emphasizing that strength outcomes across different anatomical regions represent related but distinct constructs.

Regarding aerobic capacity, the associations observed between 6 MWT performance and ergometry-derived METs are consistent with previous studies demonstrating the functional and prognostic value of the 6 MWT (Guazzi et al. [21]; Fuentes et al. [26]; Saito et al. [27]). However, the modest strength of these associations in our cohort underscores the multifactorial nature of cardiorespiratory fitness in cardiovascular populations. Unlike laboratory-based exercise testing, the 6 MWT is influenced not only by cardiovascular reserve but also by peripheral muscle function, gait efficiency, motivation, symptoms, and comorbidities. These findings reinforce the interpretation of the 6 MWT as a functional field test rather than a direct surrogate of maximal exercise capacity.

The associations between morphofunctional parameters and functional outcomes observed in this study are also supported by previous literature. Phase angle, fat-free mass, and body cell mass are recognized markers of cellular integrity, nutritional status, and metabolically active tissue, and have been consistently associated with muscle strength, functional capacity, and prognosis in clinical populations (Yokomachi et al. [28]; Ślązak et al. [29]; Acharya et al. [30]; Kowshik et al. [31]). Similarly, the relationship between rectus femoris ultrasound parameters and functional performance is consistent with emerging evidence supporting nutritional ultrasound as a valid tool for assessing muscle quantity and quality (Cruz-Jentoft et al. [32]; de Luis et al. [33]).

Nevertheless, some differences with previous studies should be acknowledged. For example, rectus femoris cross-sectional area values in our cohort were higher than those reported by Matsuo et al. [34], which may reflect differences in population characteristics, clinical stability, and measurement protocols. These discrepancies highlight the importance of contextual interpretation when comparing morphofunctional parameters across studies.

An additional relevant finding is the weak association observed between muscular strength and aerobic capacity, which is consistent with previous reports (Paluch et al. [12]). This reinforces the concept that these domains are physiologically distinct and should be assessed independently, as they reflect different underlying systems and may carry distinct prognostic implications.

From a clinical perspective, our findings suggest that simple and widely available tools such as handgrip dynamometry and the 6 MWT may provide complementary information to support functional assessment in cardiac rehabilitation. From a practical perspective, handgrip strength may offer a simple and accessible parameter to help inform resistance exercise prescription; however, given the cross-sectional design, this approach should be considered preliminary and requires prospective validation to confirm its safety and clinical applicability.

Likewise, the combined use of age, 6 MWT distance, and Borg perceived exertion score may offer a feasible approach to estimate aerobic exercise intensity when cardiopulmonary exercise testing is unavailable. However, the predictive capacity of this model was modest (R^2^ = 0.288), indicating that a substantial proportion of variability remained unexplained. Therefore, this model should be interpreted as a complementary tool rather than a standalone method for exercise prescription. In clinical practice, its use should be integrated with heart rate monitoring, perceived exertion, hemodynamic response, and clinical judgment to ensure safety.

Although handgrip strength showed a significant association with estimated quadriceps 20-RM, a substantial proportion of the variance remained unexplained (R^2^ = 0.521). This highlights the limitation of predicting lower-limb dynamic strength from upper-limb isometric measures and suggests that reliance on handgrip strength alone for lower-extremity resistance prescription may lead to imprecision. Therefore, complementary lower-limb assessments should be considered in clinical practice.

Therefore, these tools should be interpreted as part of an integrated clinical assessment rather than as standalone decision-making instruments. Prospective studies are needed to determine whether their use improves exercise prescription accuracy, safety, and clinical outcomes.

From a theoretical standpoint, this study supports the concept of physiological interrelationship between functional and morphofunctional domains, suggesting that structural and functional parameters are interrelated in clinically meaningful ways. From a practical perspective, these findings highlight the potential role of accessible, low-cost tools in bridging the gap between comprehensive assessment and real-world feasibility in cardiac rehabilitation settings.

These findings may also have potential applicability beyond outpatient cardiac rehabilitation settings, including home-based rehabilitation programs, low-resource environments, and frail or older cardiovascular populations, supporting the possible translational utility of these assessment approaches across different clinical contexts [35,36,37].

The main strengths of this study include the integration of multiple complementary assessment modalities, providing a multidimensional evaluation of nutritional, muscular, and functional status. However, several limitations must be acknowledged. The cross-sectional design precludes causal inference, the single-center setting may limit generalizability, and the absence of longitudinal follow-up prevents assessment of responsiveness to training and prognostic impact. In addition, the predictive performance of the regression models may be overestimated due to the single-cohort design and the absence of external validation.

Future research should focus on prospective validation of these approaches, including their ability to guide individualized exercise prescription, predict training response, and improve clinical outcomes across different cardiovascular phenotypes.

## 5. Conclusions

This study highlights the clinical utility of integrating simple, accessible, and reproducible functional and morphofunctional tools (specifically handgrip strength, the 6 MWT, bioelectrical impedance-derived parameters, and nutritional ultrasound) to support individualized exercise prescription within the context of cardiac rehabilitation, based on complementary functional and morphofunctional assessment rather than as standalone decision-making tools.

Handgrip strength emerged as a strong predictor of upper-limb submaximal strength, while its association with lower-limb performance was more moderate, whereas the combination of 6 MWT distance and perceived exertion provided a pragmatic but limited estimate of aerobic exercise capacity. When integrated with morphofunctional markers such as phase angle, fat-free mass, body cell mass, and rectus femoris cross-sectional area, these assessments may offer a low-cost and feasible approach to inform exercise intensity and monitor functional progression without the need for sophisticated equipment.

Overall, the moderate and context-dependent predictive performance of the proposed models and the observed physiological associations support the potential complementary clinical value of these tools in routine practice. Importantly, the weak relationship between strength and aerobic performance underscores the need to assess these domains independently when planning exercise interventions. However, given the cross-sectional design and lack of external validation, these findings should be considered exploratory and hypothesis-generating, and do not allow inference of causality or training responsiveness over time. This multidimensional strategy may contribute to more informed and individualized exercise prescription, and its potential applicability in broader settings (e.g., low-resource or ambulatory environments and diverse cardiovascular subgroups) warrants further prospective validation.

## Figures and Tables

**Table 1:** Clinical, anthropometric, and functional characteristics of the total sample and their comparison by sex.

Variable	Total (*n* = 223)	Men (*n* = 174)	Women (*n* = 49)	*p* Value
Clinical and anthropometric variables				
Age, years	59 [11]	58 [10]	61 [13]	0.207
Weight, kg	82.6 [20.2]	86.3 [19.9]	71.6 [14.5]	<0.001
Height, cm	170 [10.5]	173 [10.0]	162 [9.0]	<0.001
BMI, kg/m^2^	28.7 [5.85]	28.9 [6.10]	28.5 [5.90]	0.135
Waist circumference, cm	103 [19]	104 [18]	96.5 [13.8]	0.058
IPAQ score, MET-min/week	858 [727]	851 [751]	876 [575]	0.453
PREDIMED score	9.00 [3.00]	9.00 [3.00]	9.00 [3.00]	0.654
Morphofunctional assessment				
PhA, °	5.60 [1.30]	5.80 [1.30]	5.20 [1.20]	<0.001
Standardized phase angle	−0.640 [1.11]	−0.640 [1.11]	−0.860 [1.14]	0.376
Fat-free mass index, kg/m^2^	18.3 [11.0]	19.2 [11.4]	16.5 [6.10]	0.001
Fat mass index, kg/m^2^	8.20 [3.40]	8.30 [2.98]	8.00 [5.10]	0.574
Ultrasound assessment				
RF-CSA, cm^2^	4.81 [1.95]	5.06 [1.73]	3.49 [1.27]	<0.001
Total abdominal subcutaneous adipose tissue, cm	2.08 [1.28]	1.95 [1.25]	2.37 [1.34]	0.027
Visceral adipose tissue, cm	0.740 [0.445]	0.765 [0.455]	0.720 [0.450]	0.339
Functional assessment				
HGSmax, kg	34.0 [14.3]	37.0 [10.0]	21.0 [6.5]	<0.001
Biceps dynamometry, kg	42.5 [20.6]	46.7 [18.7]	32.5 [11.7]	<0.001
Quadriceps dynamometry, kg	46.5 [16.4]	49.0 [15.1]	38.5 [15.6]	<0.001
Biceps 20-RM, kg	4 [1]	4 [1]	2 [1]	<0.001
Quadriceps 20-RM, kg	7 [2]	8 [2.5]	6 [2]	<0.001
6 MWT distance, m	270 [90]	270 [100]	280 [85]	0.729
6 MWT speed, km/h	2.70 [1.22]	2.80 [1.30]	2.60 [0.80]	0.529
Borg perceived exertion score	12.0 [1.0]	12.0 [2.0]	12.0 [1.0]	0.439
Exercise stress test duration, min	7.16 [3.20]	7.28 [4.20]	7.00 [2.02]	0.378
Exercise stress test METs	10.0 [4.50]	10.0 [4.95]	10.0 [3.10]	0.216

Data are presented as median interquartile range (IQR). Student’s *t*-test for independent samples was used when normality was met, and the non-parametric Mann–Whitney U test was applied otherwise, according to the Shapiro–Wilk test. Abbreviations: IQR = Interquartile Range; BMI = Body Mass Index; WC = Waist Circumference; IPAQ = International Physical Activity Questionnaire; Cuest_Diet_Med Mediterranean Diet Questionnaire; cm^2^ = square centimeters; PhA = Phase Angle; SPhA = Standardized Phase Angle; FFMI = Fat-Free Mass Index; FMI = Fat Mass Index; cm = centimeters; RF-CSA: Rectus femoris Cross-sectional area; TA-SAT = Total abdominal subcutaneous adipose tissue; VAT = Visceral adipose tissue; kg = kilograms; HGSmax = maximum handgrip strength; BDS = Biceps dynamometry strength; QDS = Quadriceps dynamometry strength; 6 MWT = 6-min walk test; m = meters; Km/h = kilometers per hour; Borg = Borg scale of perceived exertion; EST = Exercise Stress Test (ergometry); min = minutes; METs: Metabolic Equivalent of Task.

**Table 2:** Linear regression models predicting 20RM performance (biceps and quadriceps) from handgrip strength.

Dependent Variable	Predictor	Estimate (B)	SE	t Value	*p* Value	R	R^2^
Biceps 20-RM	Constant	0.3454	0.3085	1.12	0.264	0.890	0.792
	Sex	−0.0806	0.1089	−0.74	0.460		
	Age	0.00035	0.0041	0.08	0.932		
	HGSmax	0.0936	0.0045	21.01	<0.001		
Quadriceps 20-RM	Constant	2.5843	0.7892	3.27	0.001	0.722	0.521
	Sex	0.0028	0.2786	0.01	0.992		
	Age	0.0051	0.0105	0.48	0.631		
	HGSmax	0.1312	0.0114	11.52	<0.001		

Multiple linear regression models were used to estimate biceps and quadriceps 20-RM. Model fit was R^2^ = 0.792 for biceps and R^2^ = 0.521 for quadriceps (n = 194). *p* values are shown in the table. Abbreviations: 20-RM, 20-repetition maximum; B, unstandardized regression coefficient; HGSmax, maximal handgrip strength; R, correlation coefficient; R^2^, coefficient of determination; SE, standard error.

**Table 3:** Linear regression model for estimating aerobic exercise capacity expressed in METs from age, sex, 6 MWT distance, and Borg perceived exertion score.

Dependent Variable	Predictor	Estimate (B)	SE	t Value	*p* Value	R	R^2^
Ergometry METs	Constant	18.4903	2.7293	6.77	<0.001	0.536	0.288
	Age	−0.0817	0.0244	−3.34	0.001		
	Sex	−0.7446	0.4898	−1.52	0.131		
	6 MWT distance	0.0078	0.0024	3.23	0.002		
	Borg perceived exertion score	−0.5348	0.1888	−2.83	0.005		

Multiple linear regression was used to estimate ergometry-derived aerobic exercise capacity (METs) from age, sex, 6 MWT distance, and Borg perceived exertion score (n = 116). The model explained 28.8% of the variance (R^2^ = 0.288). *p* values are shown in the table. Abbreviations: 6 MWT, 6 min walk test; B, unstandardized regression coefficient; METs, metabolic equivalents; R, correlation coefficient; R^2^, coefficient of determination; SE, standard error.

## Data Availability

The data presented in this study are available on reasonable request from the corresponding author. The data are not publicly available due to privacy and ethical restrictions.

## Supplementary Materials

The following supporting information can be downloaded at: https://www.mdpi.com/article/10.3390/jcm15114336/s1, Table S1: Bioimpedance and nutritional ultrasound variables according to sex; Table S2: Data on ergometry, laboratory analysis, diet, and quality of life by sex.

## Abbreviations

The following abbreviations are used in this manuscript:

Clinical and General	
CVD	Cardiovascular Disease
CR	Cardiac Rehabilitation
BMI	Body Mass Index
HR	Heart Rate
SBP	Systolic Blood Pressure
DBP	Diastolic Blood Pressure
HbA1c	Glycated Hemoglobin
HDL	High-Density Lipoprotein
LDL	Low-Density Lipoprotein
Functional Assessment	
HGS	Handgrip Strength
HGSmax	Maximum Handgrip Strength
6 MWT	Six-Minute Walk Test
20RM	20-Repetition Maximum
METs	Metabolic Equivalents
EST	Exercise Stress Test
Morphofunctional Assessment	
BIVA	Bioelectrical Impedance Vector Analysis
PhA	Phase Angle
FFM	Fat-Free Mass
BCM	Body Cell Mass
Ultrasound Assessment	
RF-CSA	Rectus Femoris Cross-Sectional Area
RF-T	Rectus Femoris Thickness
RF-CON	Contracted Rectus Femoris Thickness
Questionnaires	
IPAQ	International Physical Activity Questionnaire
HADS	Hospital Anxiety and Depression Scale
SF-8	Short Form-8 Health Survey
