# Supplementary material for: Functional Thresholds Derived from Dynamometry and 6-Minute Walk Test with Morphofunctional Assessment to Guide Individualized Exercise Prescription in Cardiac Rehabilitation
Source: J Clin Med. 2026 Jun 3;15(11):4336. doi: 10.3390/jcm15114336 (PMC13257808; doi:10.3390/jcm15114336)
Supplement: Supplementary file 1 [file jcm-15-04336-s001.zip › jcm-4299073-supplementary.pdf]

**Supplementary Table 1.** Bioimpedance and nutritional ultrasound variables. Comparison between sexes.

| Variable | Total n = 223 | Men n = 174 | Women n = 49 | p-value |
|---|---|---|---|---|
| **BIVA** | | | | |
| Rz (Ω) | 499 [122] | 471 [94.4] | 601 [112] | < **0.001*** |
| Xc (Ω) | 50.0 [14.9] | 48.4 [13.8] | 54.0 [14.0] | < **0.001*** |
| BCM (kg) | 30.4 [9.35] | 32.3 [6.80] | 21.1 [3.50] | < **0.001*** |
| NAK ratio | 1.01 [0.21] | 1.02 [0.20] | 0.96 [0.23] | 0.118 |
| Hydration (%) | 73.5 [0.70] | 73.5 [0.60] | 73.3 [0.70] | 0.068 |
| Nutrigram (mg·24h/htm) | 903 [246] | 949 [189] | 643 [126] | < **0.001*** |
| SMI (kg/m²) | 9.60 [2.25] | 10.1 [1.60] | 7.00 [1.20] | < **0.001*** |
| **Muscle Ultrasound** | | | | |
| RF-CIR (cm) | 9.57 [1.77] | 9.89 [1.69] | 8.64 [2.02] | < **0.001*** |
| RF-X-axis (cm) | 3.90 [0.885] | 3.96 [0.862] | 3.62 [0.995] | < **0.001*** |
| RF-T (cm) | 1.43 [0.530] | 1.52 [0.485] | 1.10 [0.440] | < **0.001*** |
| L-SAT (cm) | 0.720 [0.550] | 0.630 [0.372] | 1.31 [0.740] | < **0.001*** |
| RF-CON (cm) | 1.84 [0.580] | 1.99 [0.570] | 1.35 [0.430] | < **0.001*** |

Data are presented as median (interquartile range, IQR). The Student's t-test for independent samples was used when normality was met, and the non-parametric Mann–Whitney U test was applied otherwise, according to the Shapiro–Wilk test.

**Abbreviations:** IQR = Interquartile Range; BIVA = vectorial bioimpedance analysis; Rz = Resistance; Xc = Reactance; BCM = Body Cell Mass; NAK = Sodium/Potassium; mg 24h/htm = milligrams per 24 hours per square meter; SMI = Skeletal Muscle Mass Index; RF-CIR = Rectus Femoris Circumference; RF-X-axis and RF-T = Rectus Femoris Thickness (Y-axis Diameter); L-SAT = Leg adipose Subcutaneous tissue; RF-CON = Contracting Rectus Femoris Muscle.

**Supplementary Table 2.** Data on ergometry, laboratory analysis, diet, and quality of life by sex.

| Variable | Total n = 223 | Men n = 174 | Women n = 49 | p-value |
|---|---|---|---|---|
| **Functional measurement (EST)** | | | | |
| P-B HR% (%) | 77.0 [11.8] | 77.0 [11.8] | 77.5 [10.8] | 0.737 |
| SBP baseline (mmHg) | 120 [20.0] | 120 [20.0] | 120 [19.0] | 0.329 |
| DBP baseline (mmHg) | 70 [17.0] | 70 [15.0] | 70 [20.0] | 0.357 |
| SBP post (mmHg) | 139 [35.8] | 140 [32.5] | 130 [40.0] | 0.157 |
| DBP post(mmHg) | 71.0 [10.0] | 75 [10.0] | 70 [10.5] | **0.002*** |
| HR baseline (bpm) | 72.0 [22.3] | 73 [24.0] | 72 [13.0] | 0.365 |
| HR post (bpm) | 110 [54.0] | 108 [55.0] | 120 [44.3] | 0.231 |
| **Laboratory data** | | | | |
| Glucose (mg/dL) | 98.0 [20.0] | 97.5 [20.0] | 98.0 [25.0] | 0.371 |
| Glomerular filtration rate (mL/min) | 85.0 [17.5] | 85.0 [15.5] | 80.0 [21.3] | 0.166 |

| Variable | Total n = 223 | Men n = 174 | Women n = 49 | p-value |
|---|---|---|---|---|
| Total cholesterol (mg/dL) | 128 [43.5] | 128 [47.0] | 135 [39.8] | 0.381 |
| HDL (mg/dL) | 40.0 [14.0] | 40.0 [12.8] | 45.0 [15.3] | **0.023*** |
| LDL (mg/dL) | 63.0 [33.5] | 63.0 [38.8] | 62.5 [22.0] | 0.991 |
| Triglycerides (mg/dL) | 114 [67.0] | 115 [68.0] | 106 [66.3] | 0.391 |
| HbA1c (%) | 5.80 [0.675] | 5.80 [0.700] | 5.80 [0.550] | 0.485 |
| **Dietary intake** | | | | |
| Energy (kcal) | 1957 [499] | 2015 [481] | 1685 [392] | **0.017*** |
| Protein (g) | 86.0 [27.3] | 88.3 [34.4] | 73.8 [21.5] | **0.017*** |
| Carbohydrates (g) | 197 [68.5] | 198 [77.2] | 196 [29.0] | 0.445 |
| Fat (g) | 81.3 [37.0] | 84.5 [51.7] | 71.9 [27.0] | **0.039*** |
| Fiber (g) | 15.2 [8.39] | 15.2 [8.34] | 12.9 [6.96] | 0.625 |
| **Quality of Life and Depression** | | | | |
| SF-8 | 23.0 [7.00] | 23.0 [8.00] | 24.0 [5.25] | 0.842 |
| HAD 1 | 9.00 [7.00] | 9.00 [7.75] | 9.00 [6.00] | 0.717 |
| HAD 2 | 6.00 [7.00] | 6.00 [7.00] | 6.00 [6.00] | 0.821 |

Data are presented as median (interquartile range, IQR). The Student's t-test for independent samples was used when normality was met, and the non-parametric Mann–Whitney U test was applied otherwise, according to the Shapiro–Wilk test.

**Abbreviations:** EST = Exercise Stress Test; % of peak heart rate from baseline heart rate (P-B HR%); SBP = Systolic Blood Pressure; DBP = Diastolic Blood Pressure; HR = Heart Rate; HDL = High-Density Lipoprotein; LDL = Low-Density Lipoprotein; HbA1c = Glycated Hemoglobin; SF-8 = Short Form-8 Health Survey; HAD 1= Hospital Anxiety and Depression Scale – Anxiety Subscale; HAD 2 = Hospital Anxiety and Depression Scale – Depression Subscale.
